# Patient and Public Involvement Work With Parents of Children With Life‐Limiting Conditions and Bereaved Parents: A Rapid Systematic Review

**DOI:** 10.1111/hex.70120

**Published:** 2024-12-08

**Authors:** Pru Holder, Bethan Page, Julia Hackett, Sarah Mitchell, Lorna K. Fraser

**Affiliations:** ^1^ Cicely Saunders Institute of Palliative Care, Policy and Rehabilitation King's College London London UK; ^2^ Department of Health Sciences University of York York UK; ^3^ Academic Unit of Palliative Care University of Leeds Leeds UK

**Keywords:** bereaved, life limiting condition, parents, patient and public involvement

## Abstract

**Background:**

Guidance and principles for involving the public in research or service planning exist but are not specific to the needs of parents of children with life‐limiting conditions or bereaved parents.

**Aim:**

Review the evidence on involving parents of children with life‐limiting conditions and bereaved parents in research, service planning and advocacy, and use this to develop best practice guidance.

**Methods:**

Rapid review following the Cochrane Rapid Reviews Methods Group Guidance. MEDLINE and EMBASE were searched for primary studies of any design and literature/systematic reviews, and grey literature searching was conducted. Sources reporting on involving parents of children with life‐limiting conditions or bereaved parents in healthcare, research, or charity work in any setting, were included. Data were charted using the UK standards for public involvement in research (PPI). Two PPI consultation workshops were conducted with parents (*n* = 13) and healthcare professionals/charity representatives (*n* = 7).

**Results:**

Six sources were included. Four reported benefits of parental involvement and two reported burdens. In relation to best practice, two reported on the importance of inclusive opportunities, three on working together, four on support and learning, three on communications, one on impact, and one on governance. PPI consultation workshops highlighted new factors which were not present in the literature around communication and understanding the impact of involvement.

**Conclusion:**

Organisations working with this group should consider offering inclusive approaches to improve diversity, levelling power imbalances, ensuring flexibility of approach, and appropriate communication and impact.

**Patient or Public Contribution:**

The study was conducted in collaboration with 13 parents of children with life‐limiting conditions and bereaved parents, and seven palliative care professionals. The group were involved at key stages of the review and contributed to the development of the findings and conduct of the review.

## 1 Introduction

As the number of children with life‐limiting conditions increases, their needs for healthcare are also rising [1]. Increasingly their families are asked by professionals and organisations to contribute to service design, advocate for funding, take formal roles such as ambassadors or trustees and be involved in the design and conduct of research studies. Across all of health and care, public involvement activities have increased in priority in the United Kingdom (UK) and internationally. Patient and public involvement (PPI) in research is defined as research carried out with or by members of the public to, for example, offer advice as members of a project steering group, comment on and develop research materials and undertake interviews with research participants [2]. PPI in health commissioning is defined as involving service users, patients, carers and families and those with lived experience as well as the wider public and stakeholder organisations representing these networks and communities in the planning (including policy‐making and relevant programmes), buying, and monitoring of public health services [3]. PPI activities also occur in other settings beyond research and commissioning, such as parents sitting on an advisory board for a charity or healthcare improvement project or presenting at a conference or training event for professionals. The focus of this paper is on best practice for PPI with parents of children with life‐limiting conditions and bereaved parents, so it is sensitive to their specific needs and meaningful for all involved.

There are many benefits of PPI work for services, children, and families: PPI is fundamental to protect and promote the interests of families of children with life‐limiting conditions and bereaved parents, and it helps to ensure that research and service improvement is relevant and impactful. Healthcare professionals, organisations and researchers are often reluctant to involve bereaved parents and parents of children with life‐limiting conditions as they can assume PPI work would be too burdensome for these families [4, 5]. Evidence from the literature suggests that many parents are keen to support research and service improvement work, often reporting a desire to help others and give back [6]. There are challenges and risks when PPI work is not done well, including perceived tokenism and distress for families [5]. Power imbalances can exist between professionals and parents [7] and there can be difficulties involving a diverse range of families [8]. There is no doubt the input of parents is valuable, but at present there is no good quality guidance on best practice when involving parents whose child has a serious or life‐limiting condition, or when their child has died.

There is an extensive wider literature and policies on the principles of involving the public in research or service planning, but this guidance is not specific to the needs of parents of children with life‐limiting conditions or bereaved parents. A systematic review identified 65 toolkits for supporting PPI in research activities [9]. In the UK, six standards have been published on what ‘good PPI’ looks like in health and care research [10]. The standards cover six ‘values‐based’ areas: Inclusive Opportunities, Working Together, Support and Learning, Communications, Impact and Governance. Whilst these can inform effective PPI work with parents of children with life‐limiting conditions and those who are bereaved, they do not consider the unique experiences of these parents and their need for support and flexibility when participating in these activities. This includes the emotional, practical and financial implications of dealing with caring responsibilities or the death of a child. For example, whilst many PPI groups meet regularly, the fragility of the health of children with life‐limiting conditions means that short notice cancellation from parents can be necessary. There are also PPI guidelines available in relevant populations, such as in adult palliative care [11] where there are similar concerns about the burden on families of public involvement work, and a highlighted need for flexibility of approach, which is relevant to parents of children life‐limiting conditions and bereaved parents [12].

The aim of this study was to systematically review the evidence regarding the involvement of parents of children with life‐limiting conditions and bereaved parents in (i) service planning, (ii) delivery, (iii) research and (iv) advocacy or board positions (e.g., charities). The review included PPI consultation workshops with key stakeholders. This project aims to develop best practice guidance for healthcare professionals and organisations when involving parents of children with life‐limiting conditions or bereaved parents.

## 2 Methods

### 2.1 Study Design

This project emerged as a priority topic from discussions with charities and researchers working with bereaved parents and parents of children with life‐limiting conditions. A pragmatic decision was made to use a rapid review methodology based on the project's funding constraints and its appropriateness in supporting the timely synthesis of available literature to inform actionable guideline recommendations in urgent and emergent topics. This rapid review was conducted in accordance with the Cochrane Rapid Reviews Methods Group Guidance [13]. The seven stages of the review included: (i) setting the research question and topic refinement; (ii) setting eligibility criteria; (iii) searching; (iv) study selection; (v) data extraction; (vi) risk of bias assessment; and (vii) synthesis. As per the guidance, the involvement of key stakeholders was incorporated throughout the stages of the review. Two consultation workshops with key stakeholders (parents of children with life‐limiting conditions and/or bereaved parents and healthcare professionals/charity representatives) were held during the review process.

As an extension to the Preferred Reporting Items for Systematic Reviews and Meta‐Analyses (PRISMA) for rapid reviews is still underway, the rapid review report was informed by the PRISMA Checklist [14] to the extent possible and adapted accordingly. The search results including the process of identification, screening, and inclusion of articles for the rapid review were reported using the PRISMA flow chart [14]. In line with the Cochrane Rapid Reviews Methods Group Guidance an online systematic review software [15] was used for study selection and extraction. This rapid review was registered on the Open Science Framework (https://osf.io/6dkam).

### 2.2 Search Strategy

MEDLINE and EMBASE were searched from inception to 17 October 2023. The search strategy included four main concepts: ‘parents’, ‘children and young people’, ‘life‐threatening conditions’ and ‘involvement (in healthcare, research or charity work)’. The full search strategies are reported in File S1. ‘Life‐limiting’ conditions are defined as

- … those for which there is no reasonable hope of cure and from which children or young people will die. Some of these conditions cause progressive deterioration rendering the child increasingly dependent on parents and carers.
- … those for which curative treatment may be feasible but can fail, such as cancer, which are also included [16].

Inclusion and exclusion criteria are shown in Table 1.

**Table 1:** Inclusion and exclusion criteria.

**Inclusion criteria**
Focus on involving parents of children with life‐limiting conditions or bereaved parents in service planning, delivery, research, advocacy, or board positions (e.g., charities). Participants are any stakeholders who provide data in relation to involving parents of children with life‐limiting conditions or bereaved parents in healthcare or charity work. Any cultural/sub‐cultural setting and geographic location. Published primary studies of any research design. Published literature or systematic reviews (if different data are presented to sources otherwise presented in the review). Published studies from any date. Written in English language.
**Exclusion criteria**
Studies where all participants are > 18 years of age. Articles such as case studies, case series, books, editorials, commentary or opinion pieces or conference abstracts. In language other than English.

Systematic grey literature searching was conducted by two reviewers (PH and BP) and adapted from the search terms above. Grey literature searching consisted of two parts. First, targeted website searching of charities and organisations who support children with life‐limiting conditions was conducted, looking for examples and guidance on how they involve parents in service planning; delivery; research; advocacy or board positions (e.g., charities). A list of relevant organisations and websites was compiled by the research team who have extensive knowledge of the field of children's palliative care. This included SANDS, Together for Short Lives (TFSL), Council for Disabled Children (CDC), Nuffield Council. Bioethics, Royal College of Paediatrics & Child Health (RCPCH), National Bereavement Alliance, Well Child, Barnardo's, Bliss, and Contact. Second, we searched the websites of national organisations, not specific to child/palliative care, for relevant broader examples and guidance on PPI, including NHS England, HQIP National Audits, and Social care online.

Retrieved articles were imported into Covidence [15]. Titles and abstracts were screened independently by two reviewers (PH and BP). Full‐text review of potentially eligible articles was independently conducted by the same two reviewers. Disagreements were resolved with a third reviewer (LF).

### 2.3 Data Extraction

Data was extracted and verified by two reviewers (PH and BP) using a data extraction tool adapted from the Joanna Briggs Institute template [17] (File S2). Disagreements were resolved with an additional reviewer (LF).

### 2.4 Collating and Summarising Data

**Figure 1:**
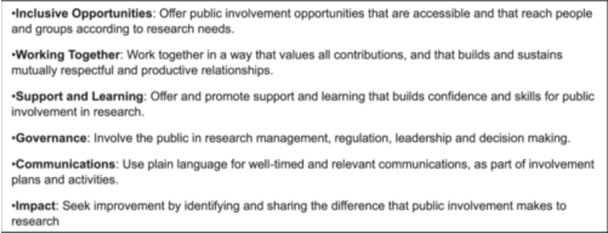
The UK Standards for Public Involvement [10].

During data collation and synthesis, the authors concluded that the nature of the findings could best be represented and organised using the six categories of the UK standards for PPI: (1) inclusive opportunities; (2) working together; (3) support and learning; (4) communications; (5) impact; and (6) governance). The research team piloted this framework on the included papers; and following discussion, it was felt the framework captured all the key themes extracted from the included papers. The framework is a well‐recognised general framework for PPI which is increasingly used in the UK context: the framework served as a useful tool for organising the extracted data, and for identifying the specifics relating to PPI in parents of children with life‐limiting conditions and bereaved parents. The UK Standards for Public Involvement [10] are described in Figure 1. Data was also extracted on the benefits and burdens of involvement. Quality appraisal of papers is not required for rapid reviews and was not conducted as per Joanne Briggs Institute guidance [17].

### 2.5 Consultation with Stakeholders

Two PPI consultation workshops with key stakeholders were held during the review process (parents of children with life‐limiting conditions, bereaved parents, and healthcare professionals and charity representatives who work with children with life‐limiting conditions and their families). Potential workshop participants were identified through existing networks and contacts of the research team but did not have a longitudinal relationship with the researchers. A diverse range of parents was purposefully included to ensure representation across the country including a mixture of fathers and mothers (fathers *n* = 2; mothers *n* = 11), with a range of experiences (bereaved parent n = 8; parent of child currently living with a life‐limiting condition n = 5). Four healthcare professionals from three NHS Trusts were recruited, including paediatric/palliative care consultants (*n* = 3) and a children's palliative care nurse (*n* = 1). Three charity professionals across the palliative care sector were also recruited. As workshop attendees were providing PPI, ethical approval was not required (as confirmed with our University Ethics team). Workshop participants were sent a detailed description of the workshop plan before agreeing to take part.

During Consultation Workshop One, members of the stakeholder group introduced themselves and started to establish rapport through informal discussion about experiences and knowledge of PPI. Initial findings from the rapid review search were presented and feedback was sought on the following: (i) the review process, (ii) views on the examples of good guidance identified from the literature search, and (iii) views on what is potentially missing from the guidance identified. During Consultation Workshop Two the following was sought: (i) feedback on what the format and content of best practice guidance should look like, and (ii) examples from stakeholder's own experiences of what has worked well when being involved with healthcare or charity work and any challenges.

Workshops were facilitated by the research team and an artist from an external company (Nifty Fox Creative) to assist with activities to engage participants. Feedback was collected directly from stakeholders via various activities on Google Jamboard and through a process known as visual storytelling which involved the live illustration of stakeholder's ideas and feedback on the guidance. Due to the sensitive nature of the topic, stakeholders were offered a debrief directly after each workshop and were provided with contact details of the research team should they have had any concerns following the workshops.

During each consultation workshop, key findings were collated and summarised by the research team. The feedback from the consultation workshops was triangulated against the findings from the literature to highlight any discrepancies between the consultation workshops and the literature.

## 3 Results

**Figure 2:**
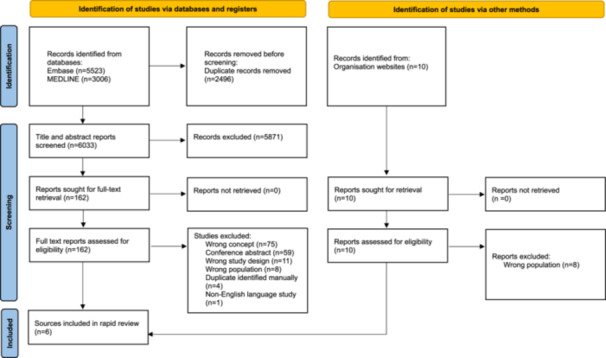
PRISMA flow chart. PRISMA, Preferred Reporting Items for Systematic Reviews and Meta‐Analyses.

Searches retrieved 8529 articles from bibliographic databases. After deduplication, 6033 papers were eligible for the title and abstract screening and 162 required full‐text assessment. An additional 10 sources were identified through grey literature searching. A total of four papers [18, 19, 20, 21] and 2 organisation resources [22, 23] were eligible for inclusion (Figure 2). Full article characteristics tables are available in File S3.

Publication dates ranged from 2013 to 2022, with the majority published after 2020 (Table 2). The primary source of data collection was qualitative methods. Primary studies and grey literature originated from four countries, with half from the UK [20, 22, 23], and the remaining from the USA [18], Australia [21], and Canada [19]. Most study participants/target groups comprised of mixed groups of families and health professionals. The majority of sources discussed parent's involvement in clinical education and quality improvement, with the remaining source considering parent's involvement in research.

**Table 2:** Source characteristics (*n* = 6).

Characteristic	*n* (%)
Year of publication	
2013–2017	2
2018–2022	3
Unknown	1
Country	
UK	3
US	1
Australia	1
Canada	1
Source design	
Qualitative methods	3
Organisation guidelines	2
Mixed methods	1
Population	
Mixed group of families and health professionals	3
Bereaved parents/caregivers	2
Mixed group of bereaved and non‐bereaved parents/caregivers	1
Condition/specialty	
Mixture of conditions	6
Type of parental involvement	
Parental involvement in healthcare/clinical education	5
Parental involvement in research (i.e. as advisors to research design)	1

Initial data extraction was carried out by extracting and grouping the benefits and burdens of parental involvement. We compared the findings from the literature to the findings from the stakeholder consultation workshops. Guidance from both these sources is presented in Table 3.

**Table 3:** Key recommendations from the published literature (*n* = 6 sources) and stakeholder consultation workshops: Benefits and burdens of involvement to parents.

Benefits and burdens	Key recommendations	PPI	Published literature *n*	Study/studies
Benefits of	Let parents heal and express grief	✓	4	[18, 19, 20, 21]
Involvement	Let parents help other families and give something back	✓	3	[18, 19, 20]
	Let parents help staff understand and improve the system	✗	2	[18, 20]
	Help parents to feel empowered (by being able to make changes to services)	✗	2	[18, 19]
	Let parents talk about their child and keep memories alive	✗	2	[18, 19]
	Provide social support for parents and opportunity to connect with others to feel understood/validated	✓	1	[19]
	Public involvement activities can help level the power between parents and healthcare professionals	✓	0	
Risks/burdens of	Understand that taking part can be upsetting for parents	✓	2	[18, 19]
involvement	Balance professionals' ability to be emotionally open without becoming emotionally overwhelmed	✗	1	[18]
	Minimise burden and fatigue on parents	✓	0	
	Keep parent's involvement short to avoid taking away valuable time that could be spent in other ways	✓	0	
	Minimise pressure on parents and sense of obligation to be there	✓	0	
	Create equal power between parents and professionals	✓	0	
	Make sure parents' involvement is not a tick box exercise	✓	0	
	Reassure parents that taking part will not affect child's care	✓	0	

*Note:* NB: ✓ indicates recommendation was proposed by stakeholders in the consultation workshops;

✗ indicates recommendation was not mentioned by stakeholders in the consultation workshops.

**Figure 3:**
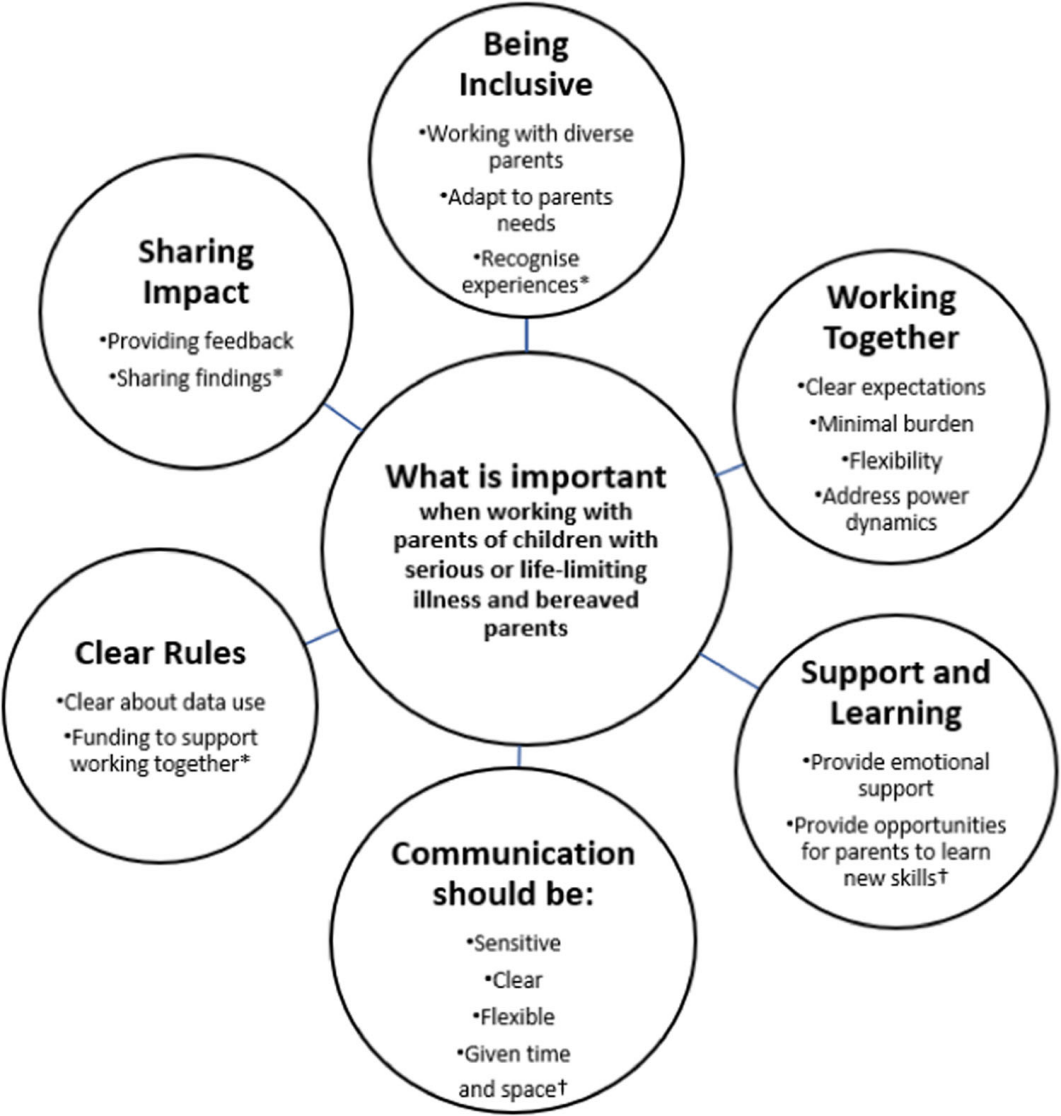
Summary of key themes from the published literature and stakeholder consultation workshops. NB: * indicates theme was derived from the stakeholder consultation workshops only and was not present in literature; † indicates theme was derived from the published literature only and was not discussed in the stakeholder consultation workshops. All other themes were derived from both sources.

Data related to best practice guidance were then extracted and categorised according to the six categories of the UK standards for PPI [10]. This allowed us to explore best practice guidance in the context of maximising the benefits and minimising the burden for parents. Guidance from both these sources is presented in Figure 3 and Table 4.

**Table 4:** Key recommendations from the published literature (*n* = 6 sources) and stakeholder consultation workshops: Based on UK Standards for PPI [10].

Level of framework	Key recommendation	Examples/description	PPI	Published literature n	Study/studies
Inclusive opportunities	Get to know the needs of the group and adapt plans to meet their needs so activities are accessible to all	*•Consider practical needs (i.e., meetings to occur during school hours, weekends, avoiding religious holidays etc)* *•Consider education level, first language, cultural and religious beliefs, and phone/computer capabilities and access* *•Consider potential grief days (i.e., infant birth or death, Mother's/Father's Day)*	✓	1	[22]
	
	Make sure you involve a diverse range of parents	*•Find ways to reach all parents whoever they are so different voices can be heard and a diverse mix of parents are represented*	✓	1	[23]
	Recognise that prior experiences of care may influence how parents contribute	• *Make sure parents do not feel like giving feedback about their involvement will impact on their relationship with clinical team who may also be involved*	✓	0	
Working together	Be clear about what parents should expect	*•Have clear criteria for who should be involved (i.e., what experience they need and potential conflicts of interest)* *•Provide a summary of involvement for each session i.e., purpose, aims, what their role will be/what is expected of them, how long the process will take, number of meetings/contact and of any delays, who the other members will be, potential risks of taking part, what the wider impact will be/what it will lead to. This could involve a pre‐engagement session, so parents know what to expect, especially those who have not done public involvement work before*.	✓	3	[19, 22, 23]
	Offer flexibility throughout – in how you approach families to take part, where an activity takes place, timing of activity	*•Be flexible in finding the right time to approach families (e.g. there should not be a cut off for how soon after a child's death to contact parents)* *•Allow parents to choose where they would like the work to take place and create a ‘safe space’ for them (i.e., home/remote/neutral location/place of employment)* *•Provide logistical information (i.e., locating and paying for parking)* *•Offer parents different ways to be involved and let them choose (i.e., option to view a recording afterwards and feedback in own time)* *•Give parents flexibility and choice depending on their schedule (i.e., option to take time out of sessions or change plans last minute)*.	✓	2	[22, 23]
	Address the power dynamic	*•Build trusting relationships and clarify that everyone has an equal part to play (e.g., consider asking parents what their motivations are for taking part and ask researchers to share something personal)* *•Provide payment, travel/food expenses (and information on how to claim) in format of parent's choice* *•Offer opportunities for parents to help lead the work i.e., sessions could be co‐chaired with parents (make sure parents are prepared and supported in the role)*.	✓	2	[19, 22]
	Minimise burden	*•Keep pre tasks and preparation to a minimum and clearly explain to parents what they need to do well in advance*.	✓	1	[22]
	Don't assume involvement will be too burdensome, always ask	*•Let parents decide whether taking part would be in their best interests, do not assume involvement would be too burdensome*.	✓	0	
Support and learning	Make sure parents are emotionally supported	*•Signpost parents to a variety of emotional and practical support options throughout (i.e., psychological support, external charities, local support groups, specific person in the research/clinical team)* *•Give parents flexibility to leave or step out without reason* *•Harness social/peer support among parents* *•Be aware of the needs of the parent and child (e.g., how unwell they are or how long ago they died) and tailor support accordingly* *•Consider paying for a professional with the relevant support skills to contact families after they have taken part*.	✓	4	[19, 21, 22, 23]
	Provide parents with opportunities to learn new skills	*•Support parents to generate new knowledge and skills (i.e., formal knowledge/research methods/share knowledge)*	✗	1	[22]
Communications	Clear communication	*•Provide information that is easy to understand in language of choice that avoids jargon but is not patronising* *•Share minutes of group sessions afterwards*. *•Provide feedback and follow up about findings/outcomes*	✓	2	[22, 23]
	Give parents space and time	*•Allow time for rapport building/time for parents to talk about child before involvement* *•Ensure pacing of information is in line with parents’ preferences and not rushed* *•Give parents opportunities to ask questions and share concerns throughout* *•Provide contact details and keep lines of communication open with parents afterwards should parents wish to get in touch*.	✗	2	[19, 23]
	Sensitive communication	*•Show compassion, respect and sensitivity when communicating with parents (i.e., consider avoiding sensitive language such as ‘end‐of‐life’)* *•Consider using charities/bereavement programme coordinators for advice* *•Maintain sensitivity and flexibility if parents digress/go off topic*.	✓	1	[23]
	Adapt communication to the needs and wishes of the parents	*•Have multiple ways to communicate with parents and give them as much choice as possible (i.e., phone/email/letter clearly marked/opt in/out cards)* *•Personalise communication (i.e., verify child's name/nickname from outset)* *•Consider acknowledging child's birthday if parent's wish*.	✓	1	[23]
Impact	Provide feedback to parents	*•Let parents know what the impact has been/what are you are doing afterwards so that they feel their contribution was worthwhile*.	✓	1	[19]
	Share findings of the project	*•Feedback and findings from the work should be shared widely* *•Parents should be included in decisions around how the project/findings will be shared*.	✓	0	
Governance	Be clear about data protection	*•Be clear how personal details and views will be kept confidential*. *•Be clear about when it is okay for health professionals/researchers to contact parents*.	✓	1	[22]
	Ensure adequate funding/provisions are in place for public involvement activities	*•This could include funding for PPI coordination and/or a PPI representative i.e., a parent to cochair sessions/involving families in design of information sheets etc*.	✓	0	

*Note:* NB: ✓ indicates recommendation was proposed by stakeholders in the consultation workshops;

✗ indicates recommendation was not mentioned by stakeholders in the consultation workshops.

### 3.1 Benefits of Involvement

The majority of the included sources described the clear benefits of PPI activities from the perspective of parents. [18, 19, 20, 21] Participating gave parents a sense of purpose, giving additional meaning to their children's lives, [18] and was seen as a way of giving back for the care they and their children received. [18, 19] Being able to share family stories and connect with others was seen as affirming, [19] and sometimes a healing experience for the parents. [18, 19, 20, 21] Their contribution was beneficial in terms of helping other families, [18, 19, 20] and helping staff understand families better and improve the system. [18, 20] An additional finding from the stakeholder consultation workshops was that PPI activities could help level the power between parents and healthcare professionals.

### 3.2 Risks and Burdens of Involvement

Papers discussed the emotional burden and challenge of sharing and reexperiencing pain for parents. [18, 19] Additional findings from the stakeholder consultation workshops were that participating in PPI activities could take valuable time away from parents that could be spent in other ways and lead to increased fatigue. They could also sometimes feel like tick‐box exercises or there could be unequal power dynamics which could be distressing to parents. Some parents felt that taking part in PPI activities with clinical teams involved in their child's care could negatively impact the subsequent care they received.

The following section describes guidance on conducting PPI activities emerging from the literature and consultation workshops, as mapped to the six UK Standards for Public Involvement categories: [10] (1) inclusive opportunities; (2) working together; (3) support and learning; (4) communications; (5) impact; and (6) governance. Four sources reported guidance on at least one of these categories [19, 21, 22, 23], with only one source reporting guidance on five categories [22] and no sources reporting guidance which spanned all six standards.

### 3.3 Inclusive Opportunities

*Equality & diversity*. Included sources highlighted the importance of involving a diverse range of parents in PPI and research activities [23] and providing parents with different ways to share views, thoughts, and concerns. [23] Activities need to be accessible to all parents, and this includes the consideration of their cultural and religious beliefs and practical needs (i.e. meetings might occur during school hours or avoid major religious holidays [22]). The stakeholder consultation workshops also highlighted the inaccessibility of written PPI materials for families who have limited literacy, and the difficulty of remote or virtual methods for families with limited computer literacy or access. Parents also discussed the importance of considering parent's prior experiences of care and treatment by healthcare professionals, which could influence how they engage with PPI activities.

### 3.4 Working Together

*Inclusion Criteria*. One source reported the importance of defining the specific criteria of the parents to be involved, and ensuring any prior experience required is clearly explained. [22] An additional finding from the stakeholder consultation workshops was the need to avoid ‘gatekeeping’ whereby professionals aim to protect parents from the burden of participation by denying them access and taking away the opportunity for these parents to make an informed decision about their participation.

*Approaches to involving families*. The importance of finding the right time to involve parents was described in the literature [23] and reiterated in the stakeholder consultation workshops. This included allowing enough time after a bereavement to minimise the emotional burden for parents whilst not leaving it so long that parent's ability to recall how they felt and what they needed was lost. Parents felt there should be no pre‐defined limits to the timing of participation, and this should be flexible and dependent on each parent's experience and preferences. Parents also highlighted the importance of giving them a choice in how they would like to be contacted. For example, some parents reported that receiving a letter could be the least intrusive contact method in comparison to a direct or ‘cold’ telephone call and allow them time to carefully think about their involvement. However, being mindful of the individuality of parent's preferences was paramount. It may also be advantageous to consider who is making the first contact with parents and what impact this can have (i.e. in terms of familiarity and trust).

*Agreeing to participate*. Parents in the consultation workshops discussed the importance of incorporating flexibility (i.e. giving parents time to consider their decision to participate) and using unintrusive methods such as an ‘opt‐in’ card. The importance of overcoming barriers to involving parents such as managing their preconceptions towards PPI and the usefulness of training/education for professionals were also discussed.

*Logistical issues*. Parents in the stakeholder consultation workshops discussed the importance of ensuring flexibility for parents in terms of the location and setting of participation. For example, some parents may find the hospital setting triggering and value the emotional comfort, privacy and convenience offered by the home environment, others may find the home too intrusive and would prefer a neutral location (i.e. private office space or place of employment). The importance of allowing parents to schedule their involvement around their family needs, employment, and travel time, whilst also providing logistical information (i.e., locating and paying for parking) was also discussed.

*Type of involvement/duration*. The literature discussed the importance of preparing parents about what their involvement would entail, such as, the amount of meetings/contact per year, the long‐term timeline, and who the other members of the group/committee would be. [19, 22, 23] Parents were likely to decline to participate if they see the methods as too involved or burdensome and using up valuable time that could be spent in other preferred ways. Therefore, any preparation required should be well explained to parents before taking part and overall time‐requirement kept to a minimum. [22] There was limited literature on preferred methods of participation, although the importance of offering parents different ways to be involved was reported [23]. Additionally, the stakeholder consultation workshops highlighted the importance of creating trusting relationships and ensuring an equal power balance between parents and professionals (e.g. asking researchers to share something personal about themselves or offering parents opportunities to cochair sessions).

*Payment and thank yous*. Both the stakeholder consultation workshops and literature discussed the importance of considering incentives for parental involvement such as vouchers, travel expenses including parking, overnight accommodation, childcare and training expenses and also providing clear details about how any payments can be claimed. [19, 22]

### 3.5 Support and Learning

*Support for families*. The literature discussed the importance of ensuring a variety of options are available to parents who may be negatively affected by participating, including support from the clinical/research team as well as external organisations such as local or psychological support groups and charities. [19, 21, 22] Support should be tailored, accounting for the specific needs of the parent and child (e.g., how unwell the child is/how long ago they died [23]) and allow parents the flexibility to leave without reason. [22] Additional findings from the stakeholder consultation workshops included harnessing social and peer support among parents and recruiting a professional with the relevant support skills to contact families after they have taken part.

*Opportunities for learning*. One source mentioned the value of parental participation in supporting families to learn about new research, generate new knowledge and gain new skills. [22]

### 3.6 Communications

*Communication with families*. The literature described the importance of correct pacing of information including incorporating additional time to build rapport with parents so that parents don't feel hurried, [23] and personalising communication (i.e., using the child's name from the outset [23]). Allowing parents several opportunities to ask questions and share concerns during participation and keeping lines of communication open should parents wish to get in touch after participation was seen as important. [19, 23] Additional findings from the stakeholder consultation workshops included maintaining sensitivity and flexibility if parents digress/go off topic during participation and sharing the minutes of group sessions after participation. Consulting with trained health professionals (i.e., bereavement coordinators) for advice about all aspects of communication was reported as advantageous.

*Language usage*. Sensitive language when communicating with parents that avoid highly charged terms such as ‘end‐of‐life care’, which may cause distress and provoke negative feelings, [23] as well as clear language that avoids jargon [22, 23] was described as optimal in the literature and reiterated in the stakeholder consultation workshops.

### 3.7 Impact

*Sharing of findings*. There was limited literature on the theme of impact, although the importance of informing parents about the outcomes and impact of the work so that they feel their contribution was worthwhile was described in one source. [19] This was reiterated in the stakeholder consultation workshops, whereby parents felt strongly that the findings from work they are involved in should be shared widely, and that they should be included in decisions around how the findings are shared.

### 3.8 Governance

*Data protection*. The literature reported that parents should be aware of confidentiality and privacy of information, how other people's personal experiences should be respected and what can be shared with family/friends after their involvement. [22] Additional findings from the stakeholder consultation workshops included the importance of clear guidance for professionals and researchers about when they can contact parents.

*Funding*. Parents and professionals in the stakeholder consultation workshops discussed the importance of securing adequate funding for PPI activities or PPI representatives.

**Figure 4:**
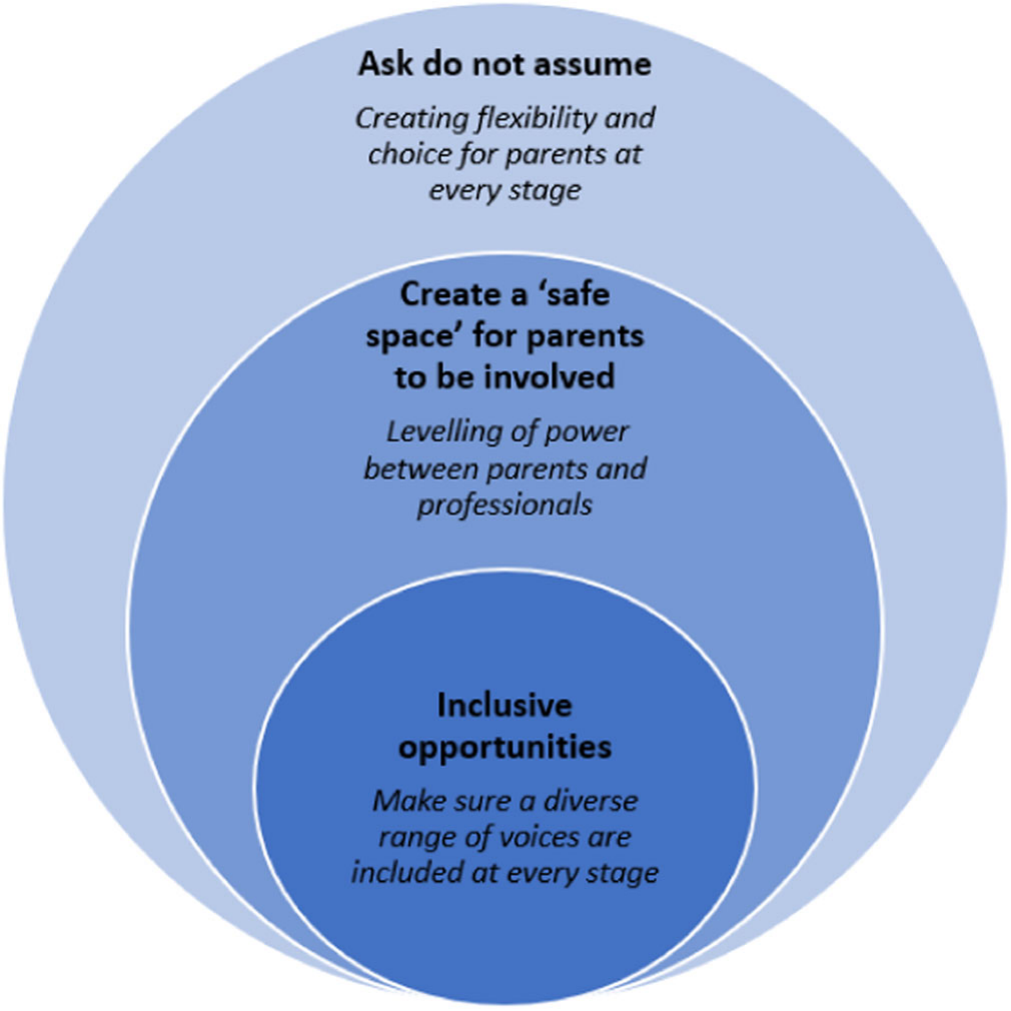
Summary of cross‐cutting themes.

Through combining the findings from the literature with our stakeholder workshops we identified cross‐cutting themes to understand what mattered most to parents (Figure 4).

## 4 Discussion

### 4.1 Main Findings

This review has identified a small volume of literature describing how to conduct PPI work with parents of children with life‐limiting conditions and bereaved parents, [18, 19, 20, 21, 22, 23] with findings evident across all categories of the UK Standards for Public Involvement. [10] Whilst a range of important factors when engaging this group are presented in the published literature (i.e., the importance of flexibility of approach [22, 23]), this review highlights a paucity of literature specific to PPI with this population. The stakeholder consultation workshops highlighted additional factors which were less well described in the literature (i.e., the importance of communicating impact and change [19]).

### 4.2 What This Study Adds

Whilst there are existing reviews on how to conduct PPI in related fields (i.e. adult palliative care, [11] children and young people, [24, 25] and general health research [9]), as well as on the recruitment and use of parents of children with life‐limiting conditions or bereaved parents as research participants, [26, 27] to our knowledge, this is the first review to focus specifically on PPI with this population. These parents require distinct consideration by researchers as they are a particularly vulnerable and underserved group with unique experiences and a need for support and flexibility due to the emotional, practical and financial implications of dealing with caring responsibilities or the death of a child. The findings of this review have been used to provide much needed best practice guidance for professionals on how to conduct PPI work with this group of parents which incorporates their unique experiences and needs.

Findings from the literature and consultation workshops demonstrated that involving parents in PPI activities can have clear benefits for parents and for services, [18, 19, 20, 21] however, when not done well, these activities can be burdensome, upsetting and potentially harmful for families. [18, 19] Literature on general patient populations outline the generalised benefits of involvement in healthcare research for patients such as self‐fulfilment and the opportunity to improve healthcare and learn [28]. Similarly, evidence from the included studies and our consultation workshops illustrate that parents often want to participate in PPI and research activities to give back [18, 19] and help other families. [18, 19, 20] The review also highlights clear personal benefits that are unique to this group of parents, such as a chance to heal, [18, 20, 21] talk about their child, [18, 19] and access social support from working together with other parents. [19, 29] Contrary to this, previous research in the field of adult palliative care, has shown there exists a reluctance among professionals to undertake involvement activities, and myths still perpetuate that patients/carers do not want to be involved, or that activities would be too upsetting. [5, 11] It is critical that parents are given the opportunity to decide whether taking part would be in their best interests and that PPI activities are designed well so they are meaningful to all involved.

Key recommendations from the literature and consultation workshops regarding what parents want from PPI activities were mapped to the six UK standards for PPI: [10] 1) inclusive opportunities; 2) working together; 3) support and learning; 4) communications; 5) impact; and 6) governance. The most commonly reported standards in the literature included ‘support and learning’, ‘working together’, and ‘communication’. Through combining the findings from the literature with our consultation workshops we identified cross‐cutting themes in effective PPI. This included the building of meaningful relationships, facilitated by clear [22, 23] and sensitive [23] communication, as well as the creation of inclusive environments which offer flexibility [22, 23] and take into account different parents' needs. [22] Whilst similar findings have been previously reported in general populations (i.e., including a diverse range of voices [30] and addressing power imbalances [31]) and in the field of adult palliative care (i.e., the importance of flexibility of approach [12]), the findings from this review highlight factors unique to this group of parents that require distinct consideration. This includes the recognition of their unique prior experiences of care and the impact this may have on how these parents contribute.

The findings from the stakeholder consultation workshops were helpful to enable us to prioritise what was most important to parents. Key considerations emphasised by parents in the consultation workshops included: (i) feeling valued and supported, (ii) understanding the purpose of the PPI activity, and (iii) having the impact clearly communicated to them. Whilst the first two considerations are well reported in the literature (i.e., feeling valued and supported [19, 21, 22, 23] and understanding the purpose of participation [19, 22, 23]) we found a relatively small amount of literature on ‘impact and change’. [19] Furthermore, considerations highlighted in the literature including providing parents with opportunities to learn new skills were not prioritised by parents.

Future work is needed to test the presented recommendations in specific contexts so that they can be tailored to the type of parental involvement and accompanied with real‐world examples. It would also be helpful to understand the strategies and resources required to do meaningful PPI in this area, and how to support wider national and international development of public involvement in children's palliative care. It is critical that PPI activities are evaluated to build on the evidence base. Further research is needed to explore important aspects such as equality, diversity, and inclusion in this population of parents, which has recently been explored in adult palliative care. [32]

### 4.3 Strengths and Limitations of the Study

The review was carried out using a registered protocol and followed best practice guidance on rapid systematic reviews. Grey literature searching was conducted, and the studies were screened by two reviewers against an inclusion/exclusion criterion, with findings systematically categorised using an existing PPI framework (the UK standards). [10] The review involved a diverse mix of parents in our stakeholder consultation workshops, including fathers as well as mothers, bereaved parents and parents whose child is living with a life‐limiting condition, and parents from minoritised ethnic backgrounds. However, far fewer fathers took part compared with mothers. Due to the under‐representation of male perspectives in research on children with life‐limiting conditions [33], it would have been useful to consider specific strategies for involving fathers as part of this work. The stakeholder consultation workshops were strengthened by the fact that researchers built in time for rapport building and debriefing, and a culture was created whereby parents felt they could contribute and feedback openly about what could be improved.

Limitations of the study include the inclusion of only English language studies. Furthermore, whilst the search was systematic, as with all rapid review methods, the completeness of searching of both the academic databases and grey literature was limited by time constraints, so it is likely that not every possible source was captured. Participants in the stakeholder consultation workshops were identified through existing networks and contacts of the research teams. This method of recruitment may have potentially biased some of the findings, however, could also have been advantageous in facilitating trust and willingness of stakeholders to engage in discussions.

### 4.4 Implications for Practice, Theory, or Policy

The findings from the consultation workshops and the review search have been used to facilitate much needed best practice guidance on how to conduct PPI work with parents of children with life‐limiting conditions and bereaved parents. Following the research, three outputs were co‐produced with the stakeholder group, including:

iv. an animation describing best practice when involving parents in research and charity work (https://vimeo.com/973314453),

v. a leaflet summarising key points to consider when involving parents in research and charity work (https://view.genially.com/668d58f75c3a0a0014797f3f),

vi. actionable guidance when involving parents in two common scenarios (parents as advisory board members and parents as presenters at conferences; https://cdn.prod.website-files.com/6492b804c6a08b5916da8df2/66c749d1e5ae950d36b0789b_Worked%20Examples_Co-working%20with%20parents.pdf).

These tools are currently being disseminated as widely as possible (i.e. via professional networks, charities, conferences). They will be vital for researchers, healthcare and charity professionals to ensure PPI work with these parents is sensitive to their specific needs and is meaningful for all involved. It will also ensure parents feel valued and supported and will facilitate the upskilling of professionals, so they feel more confident when working with this group of parents.

## 5 Conclusions

Although some considerations of involving parents of children with life‐limiting conditions and bereaved parents in PPI are reported in the literature (i.e. the importance of support), some factors identified in stakeholder consultation workshops which are most important to parents (i.e., to have the impact clearly communicated to them) are currently not prominent in the literature. All organisations undertaking PPI with this parent group should ensure: (i) a diverse range of voices is included, (ii) the power balance between parents and professionals is levelled, (iii) there is flexibility and choice for parents at every stage. If done well then this will enable parents to feel valued and supported, understand the purpose of the PPI activity, and have the impact clearly communicated to them.

## Author Contributions

**Pru Holder:** formal analysis; writing–original draft; writing–review & editing; methodology; data curation; project administration. **Bethan Page:** writing–original draft; writing–review & editing; formal analysis; methodology; data curation; project administration. **Julia Hackett:** conceptualisation; writing–review & editing; writing–original draft; methodology; funding acquisition. **Sarah Mitchell:** conceptualisation; writing–original draft; writing–review & editing; methodology; funding acquisition. **Lorna K Fraser:** conceptualisation; writing–original draft; writing–review & editing; formal analysis; methodology; data curation; project administration; funding acquisition.

## Ethics Statement

Ethical approval was not required for this review as data used for analysis was extracted from published studies and consultation workshops were classed as PPI activities.

## Conflicts of Interest

The authors declare no conflicts of interest.

## Supporting information

Supporting information.

Supporting information.

Supporting information.

## Data Availability

All data relevant to the study are included in the article or uploaded as online supplemental information.

## References

[1] L. K. Fraser , D. Gibson‐Smith , S. Jarvis , P. Norman , and R. C. Parslow , “Estimating the Current and Future Prevalence of Life‐Limiting Conditions in Children in England,” Palliative Medicine 35 (2021): 1641–1651, 10.1177/0269216320975308. 33323043 PMC8532217

[2] National Institute for Health and Care Research. About Public Involvement, https://www.peopleinresearch.org/public-involvement/(accessed 24/04/2024).

[3] NHS England. Framework for Patient and Public Participation in Public Health Commissioning, https://www.england.nhs.uk/wp-content/uploads/2017/01/ph-participation-frmwrk.pdf (2017, accessed 24/04/2024).

[4] J. C. Crocker , E. Beecham , P. Kelly , et al., “Inviting Parents to Take Part in Paediatric Palliative Care Research: A Mixed‐Methods Examination of Selection Bias,” Palliative Medicine 29 (2015): 231–240, 10.1177/0269216314560803. 25519146 PMC4361415

[5] D. Tomlinson , U. Bartels , E. Hendershot , J. Constantin , G. Wrathall , and L. Sung , “Challenges to Participation in Paediatric Palliative Care Research: A Review of the Literature,” Palliative Medicine 21 (2007): 435–440, 10.1177/0269216307077173. 17901103

[6] R. Steele , S. Cadell , H. Siden , G. Andrews , T. Smit Quosai , and L. Feichtinger , “Impact of Research Participation on Parents of Seriously Ill Children,” Journal of palliative medicine 17 (2014): 788–796, 10.1089/jpm.2013.0529. 24871891

[7] L. Locock , A. M. Boylan , R. Snow , and S. Staniszewska , “The Power of Symbolic Capital in Patient and Public Involvement in Health Research,” Health Expectations 20 (2017): 836–844, 10.1111/hex.12519. 27885770 PMC5600212

[8] J. Reynolds , M. Ogden , and R. Beresford , “Conceptualising and Constructing ‘Diversity’ Through Experiences of Public and Patient Involvement in Health Research,” Research Involvement and Engagement 7 (2021): 53, 10.1186/s40900-021-00296-9. 34294162 PMC8295976

[9] T. Greenhalgh , L. Hinton , T. Finlay , et al., “Frameworks for Supporting Patient and Public Involvement in Research: Systematic Review and Co‐Design Pilot,” Health Expectations 22 (2019): 785–801, 10.1111/hex.12888. 31012259 PMC6737756

[10] National Institute for Health and Care Research. UK Standards for Public Involvement in Research., https://sites.google.com/nihr.ac.uk/pi-standards/home?pli=1 (2019, accessed October 4, 2023).

[11] E. Chambers , C. Gardiner , J. Thompson , and J. Seymour , “Patient and Carer Involvement in Palliative Care Research: An Integrative Qualitative Evidence Synthesis Review,” Palliative Medicine 33 (2019): 969–984, 10.1177/0269216319858247. 31250702 PMC6691598

[12] H. Johnson , M. Ogden , L. J. Brighton , et al., “Patient and Public Involvement in Palliative Care Research: What Works, and Why? A Qualitative Evaluation,” Palliative Medicine 35 (2021): 151–160, 10.1177/0269216320956819. 32912087 PMC7797607

[13] C. Garritty , G. Gartlehner , B. Nussbaumer‐Streit , et al., “Cochrane Rapid Reviews Methods Group Offers Evidence‐Informed Guidance to Conduct Rapid Reviews,” Journal of Clinical Epidemiology 130 (2021): 13–22, 10.1016/j.jclinepi.2020.10.007. 33068715 PMC7557165

[14] M. J. Page , J. E. McKenzie , P. M. Bossuyt , et al., “The Prisma 2020 Statement: An Updated Guideline for Reporting Systematic Reviews,” BMJ 372 (2021): n71, 10.1136/bmj.n71. 33782057 PMC8005924

[15] Covidence Systematic Review Software. Veritas Health Innovation., (2023).

[16] Together for Short Lives. Categories of Life‐limiting Conditions, https://www.togetherforshortlives.org.uk/changing-lives/supporting-care-professionals/introduction-childrens-palliative-care/categories-of-life-limiting-conditions/(accessed October 4, 2023).

[17] JBI. JBI Manual for Evidence Synthesis, https://synthesismanual.jbi.global (2020, accessed October 4, 2023).

[18] G. Adams , A. Green , S. Towe , and A. Huett , “Bereaved Caregivers As Educators in Pediatric Palliative Care: Their Experiences and Impact,” Journal of palliative medicine 16 (2013): 609–615, 10.1089/jpm.2012.0475. 23725232 PMC3667424

[19] C. J. Bourque , S. Dahan , G. Mantha , M. Reichherzer , and A. Janvier , “My Child's Legacy: A Mixed Methods Study of Bereaved Parents and Providers' Opinions about Collaboration With Nicu Teams in Quality Improvement Initiatives,” BMJ open 10 (2020): e034817, 10.1136/bmjopen-2019-034817. PMC7476470 32895262

[20] J. Spalding and S. Yardley , “The Nice Thing About Doctors Is That You Can Sometimes Get a Day Off School’: An Action Research Study to Bring Lived Experiences From Children, Parents and Hospice Staff Into Medical Students' Preparation for Practice,” BMJ supportive & palliative care 6 (2016): 459–464, 10.1136/bmjspcare-2015-001080. 27208813

[21] S. Vemuri , J. O'Neill , J. Hynson , and L. Gillam , “Informing Simulation Design: A Qualitative Phenomenological Study of the Experiences of Bereaved Parents and Actors,” Simulation in Healthcare: The Journal of the Society for Simulation in Healthcare 18 (2023): 75–81, 10.1097/SIH.0000000000000634. 35081089

[22] Bliss. Public Involvement Role Description Template, https://view.officeapps.live.com/op/view.aspx?src=https%3A%2F%2Fs3.eu-west-2.amazonaws.com%2Fsr-bliss%2Fimages%2FPublic-Involvement-Role-Description-Template.docx&wdOrigin=BROWSELINK (accessed 17/10/2023).

[23] Sands. Sands 6 Principles of Parent Engagement in Review. Best Practice, https://www.sands.org.uk/sites/default/files/Sands_BestPractice_Sept21_Digital.pdf (2021, accessed October 17, 2023).

[24] A. Rouncefield‐Swales , J. Harris , B. Carter , L. Bray , T. Bewley , and R. Martin , “Children and Young People's Contributions to Public Involvement and Engagement Activities in Health‐Related Research: A Scoping Review,” PloS one 16 (2021): e0252774, 10.1371/journal.pone.0252774. 34106978 PMC8189547

[25] F. van Schelven , H. Boeije , V. Mariën , and J. Rademakers , “Patient and Public Involvement of Young People With a Chronic Condition in Projects in Health and Social Care: A Scoping Review,” Health Expectations 23 (2020): 789–801, 10.1111/hex.13069. 32372423 PMC7495073

[26] D. Tomlinson , U. Bartels , E. Hendershot , J. Constantin , G. Wrathall , and L. Sung , “Challenges to Participation in Paediatric Palliative Care Research: A Review of the Literature,” Palliative Medicine 21 (2007): 435–440. 17901103 10.1177/0269216307077173

[27] M. S. Weaver , K. Mooney‐Doyle , K. P. Kelly , et al., “The Benefits and Burdens of Pediatric Palliative Care and End‐Of‐Life Research: A Systematic Review,” Journal of palliative medicine 22 (2019): 915–926, 10.1089/jpm.2018.0483. 30835596 PMC6755658

[28] T. L. McCarron , T. Noseworthy , K. Moffat , et al., “Understanding the Motivations of Patients: A Co‐Designed Project to Understand the Factors Behind Patient Engagement,” Health Expectations 22 (2019): 709–720, 10.1111/hex.12942. 31379094 PMC6737762

[29] A. E. Butler , H. Hall , and B. Copnell , “Bereaved Parents' Experiences of Research Participation,” BMC palliative care 17 (2018): 122, 10.1186/s12904-018-0375-4. 30404631 PMC6223065

[30] L. Ryan , R. Wenke , J. Carlini , et al., “Exploring Barriers and Solutions to Consumer Involvement in Health Service Research Using a Nominal Group Technique,” Research Involvement and Engagement 10 (2024): 72, 10.1186/s40900-024-00604-z. 38992779 PMC11241927

[31] J. Ocloo , S. Garfield , B. D. Franklin , and S. Dawson , “Exploring the Theory, Barriers and Enablers for Patient and Public Involvement Across Health, Social Care and Patient Safety: A Systematic Review of Reviews,” Health Research Policy and Systems 19 (2021): 8, 10.1186/s12961-020-00644-3. 33472647 PMC7816359

[32] S. Mitchell , N. Turner , K. Fryer , et al., “A Framework for More Equitable, Diverse, and Inclusive Patient and Public Involvement for Palliative Care Research,” Research Involvement and Engagement 10 (2024): 19, 10.1186/s40900-023-00525-3. 38331966 PMC10851547

[33] V. Fisher , L. Fraser , and J. Taylor , “Experiences of Fathers of Children With a Life‐Limiting Condition: A Systematic Review and Qualitative Synthesis,” BMJ Supportive & Palliative Care 13 (2023): 15–26, 10.1136/bmjspcare-2021-003019. PMC9985706 34140322

