# Correction to “Patient and Public Involvement Work With Parents of Children With Life‐Limiting Conditions and Bereaved Parents: A Rapid Systematic Review”

**DOI:** 10.1111/hex.70802

**Published:** 2026-08-03

Holder, P., Page, B., Hackett, J., Mitchell, S. and Fraser, L.K. (2024), Patient and Public Involvement Work With Parents of Children With Life‐Limiting Conditions and Bereaved Parents: A Rapid Systematic Review. *Health Expectations*, 27: e70120. https://doi.org/10.1111/hex.70120.

In Section 4.4, “Implications for Practice, Theory, or Policy,” the URL provided in line “iv” was inadvertently placed. The correct URL is as follow: https://gumlet.tv/watch/69cd40681763b418bff88f9c/.

We apologise for this error.

