# Supplementary material for: Patient and Public Involvement Work With Parents of Children With Life‐Limiting Conditions and Bereaved Parents: A Rapid Systematic Review
Source: Health Expect. 2024 Dec 8;27(6):e70120. doi: 10.1111/hex.70120 (PMC11625871; doi:10.1111/hex.70120)
Supplement: Supplementary file 1 — Supporting information. [file HEX-27-e70120-s001.pdf]

Supplementary File 1: Search strategies

Medline (ovid) search strategy

**Concept**	**Search terms**
Parents/caregivers	1. parent*.tw.<br>2. caregiver*.tw.<br>3. mother*.tw.<br>4. father*.tw.<br>5. carer*.tw.<br>6. family.tw.<br>7. families.tw.<br>8. exp Parents/
Children and young people	9. P?ediatric*.tw.<br>10. child*.tw.<br>11. infant*.tw.<br>12. toddler*.tw.<br>13. adolescen*.tw.<br>14. teen*.tw.<br>15. youth*.tw.<br>16. juvenile*.tw.<br>17. neonat*.tw.<br>18. newborn*.tw.<br>19. new born*.tw.<br>20. baby.tw.<br>21. babies.tw.<br>22. boy*.tw.<br>23. girl*.tw.<br>24. minors.tw.<br>25. exp Pediatrics/<br>26. exp Child/<br>27. exp Infant/<br>28. exp Infant, Newborn/<br>29. exp Child, Preschool/<br>30. exp Adolescent/<br>31. exp Minors/
Bereaved/ life-limiting conditions	32. terminal illness*.tw.<br>33. life limit*.tw.<br>34. life threaten*.tw.<br>35. bereaved.tw.<br>36. palliat*.tw.<br>37. terminal care.tw.<br>38. hospice*.tw.<br>39. support care.tw.<br>40. exp Terminal Care/<br>41. exp "Hospice and Palliative Care Nursing"/<br>42. exp Palliative Care/<br>43. exp Hospices/
Involvement (in healthcare and charity work)	44. involv*.tw.<br>45. engag*.tw.<br>46. participat*.tw.<br>47. collaborat*.tw.<br>48. inclusion.tw.<br>49. partnership*.tw.<br>50. coproduc*.tw.<br>51. co-produc*.tw.<br>52. cocreat*.tw.<br>53. co-creat*.tw.<br>54. codesign.tw.<br>55. co-design.tw.
	56. or/1-8

	57. or/9-31<br>58. or/32-43<br>59. or/44-55<br>60. 56 and 57 and 58 and 59

Embase (Ovid) search strategy

**Concept**	**Search terms**
Parents/caregivers	1. parent*.tw.<br>2. caregiver*.tw.<br>3. mother*.tw.<br>4. father*.tw.<br>5. carer*.tw.<br>6. family.tw.<br>7. families.tw.<br>8. exp Parents/
Children and young people	9. P?ediatric*.tw.<br>10. child*.tw.<br>11. infant*.tw.<br>12. toddler*.tw.<br>13. adolescen*.tw.<br>14. teen*.tw.<br>15. youth*.tw.<br>16. juvenile*.tw.<br>17. neonat*.tw.<br>18. newborn*.tw.<br>19. new born*.tw.<br>20. baby.tw.<br>21. babies.tw.<br>22. boy*.tw.<br>23. girl*.tw.<br>24. minors.tw.<br>25. exp Pediatrics/<br>26. exp Child/<br>27. exp Infant/<br>28. exp Infant, Newborn/<br>29. exp Child, Preschool/<br>30. exp Adolescent/<br>31. exp Minors/
Bereaved/ life-limiting conditions	32. terminal illness*.tw.<br>33. life limit*.tw.<br>34. life threaten*.tw.<br>35. bereaved.tw.<br>36. palliat*.tw.<br>37. terminal care.tw.<br>38. hospice*.tw.<br>39. support care.tw.<br>40. exp Terminal Care/<br>41. exp "Hospice and Palliative Care Nursing"/<br>42. exp Palliative Care/<br>43. exp Hospices/<br>44. exp palliative nursing/<br>45. exp palliative therapy/<br>46. exp hospice care/<br>47. exp hospice nursing/
Involvement (in healthcare and charity work)	48. involv*.tw.<br>49. engag*.tw.<br>50. participat*.tw.<br>51. collaborat*.tw.<br>52. inclusion.tw.<br>53. partnership*.tw.

54. coproduc*.tw.
55. co-produc*.tw.
56. cocreat*.tw.
57. co-creat*.tw.
58. codesign.tw.
59. co-design.tw.

60. or/1-8
61. or/9-31
62. or/32-47
63. or/48-59
64. 60 and 61 and 62 and 63
