# Supplementary material for: Patient and Public Involvement Work With Parents of Children With Life‐Limiting Conditions and Bereaved Parents: A Rapid Systematic Review
Source: Health Expect. 2024 Dec 8;27(6):e70120. doi: 10.1111/hex.70120 (PMC11625871; doi:10.1111/hex.70120)
Supplement: Supplementary file 2 — Supporting information. [file HEX-27-e70120-s002.pdf]

Supplementary File 2: Data extraction tool

**Evidence source Details and Characteristics**
Citation details *(first author, year of publication, title, journal)*
Country of origin
Context
Study Aims
Study Design (*i.e. prospective, retrospective, longitudinal or cohort study*)
Research Methodology & Methods
Participant Details *(i.e. stakeholder group, sample size)*
**Details/Results extracted from source of evidence (in relation to the concept of the rapid review)**
Benefits and/or burdens of involving or engaging parents of children with life-limiting illness or bereaved parents in healthcare and charity work
Guidance/ recommendations for involving or engaging parents of children with life-limiting illness or bereaved parents in healthcare and charity work, categorised under the following headings:<br>• Inclusive Opportunities<br>• Working Together<br>• Support And Learning<br>• Communications<br>• Impact<br>• Governance
