# Supplementary material for: Patient and Public Involvement Work With Parents of Children With Life‐Limiting Conditions and Bereaved Parents: A Rapid Systematic Review
Source: Health Expect. 2024 Dec 8;27(6):e70120. doi: 10.1111/hex.70120 (PMC11625871; doi:10.1111/hex.70120)
Supplement: Supplementary file 3 — Supporting information. [file HEX-27-e70120-s003.docx]

Supplementary File 3: Article Characteristics

| **Authors, year, country** | **Aim(s)/purpose** | **Type of parental involvement** | **Methodology** | **Study population, setting** |
| --- | --- | --- | --- | --- |
| Adams et al., 2013, USA^18^ | To study motivations, expectations, challenges, benefits, and meaning making for bereaved parents involved in paediatric palliative care education of health care professionals and to learn about the challenges and possible benefits for the health care professionals | Clinical education | Interviews | Bereaved parents (n=9); healthcare professionals (n=11);  Paediatric hospital in the USA |
| Bourque et al., 2020, Canada^19^ | To describe the ongoing involvement and the perspectives of bereaved parents engaged in different types of activities in neonatal intensive care units and providers who work with them | Clinical | Mixed methods: field documentation and questionnaires | Bereaved parents (n=8); healthcare professionals (n=16);  Paediatric hospital in Canada |
| Spalding et al., 2016, UK^20^ | To explore perceptions of what medical students must learn to become ‘good doctors’ among children, parents and staff in a hospice. To collaborate with children/parents and staff to develop educational materials based on their lived experiences for medical students. To assess feasibility of student-led action research in a children’s hospice to develop research skills. | Clinical education | Mixed methods: Focus groups, interviews and activity workshops | Children (n=7); parents (n=5); staff (n=6);  Children’s hospice in the UK |
| Vemuri et al., 2022, Australia^21^ | To explore the impact and experiences of bereaved parents and actors who participated in simulation design | Clinical education | Phenomenological study: workshop and interviews | Bereaved parents (n=5); actors (n=2);  Paediatric palliative care program in Australia |
| **Grey literature** | | | | |
| Bliss, UK^22^ | Public Involvement Role Description Template | Research | Guidance | Bereaved and non-bereaved parents |
| Sands, 2021, UK^23^ | Principles of Parent Engagement in Review. Best Practice | Clinical | Guidance developed following a survey with parents (n=314) | Bereaved parents |
